# Efficacy of Bispecific Antibodies in Relapsed or Refractory Multiple Myeloma With Extramedullary Disease: A Systematic Review and Meta-Analysis

**DOI:** 10.7759/cureus.111438

**Published:** 2026-06-24

**Authors:** Oscar J Burke, Nicolas Peruzzo, Nimra Tul Ain Khan, Pragnan Kancharla

**Affiliations:** 1 Internal Medicine, MedStar Union Memorial Hospital, Baltimore, USA; 2 Hematology and Medical Oncology, MedStar Franklin Square Medical Center, Baltimore, USA

**Keywords:** bcma, bispecific antibody, gprc5d, multiple myeloma, multiple myeloma with extramedullary disease, objective response rate, relapsed and refractory multiple myeloma, systematic review and meta-analysis, talquetamab, teclistamab

## Abstract

Extramedullary disease (EMD) in multiple myeloma refers to soft-tissue plasmacytomas that spread hematogenously and grow independently of bone, an aggressive phenotype that has been associated with poorer responses and shorter survival across successive treatment eras. Bispecific antibodies are highly active in relapsed or refractory multiple myeloma (RRMM), but their efficacy in patients with baseline EMD has not been quantitatively synthesized. We performed a systematic review and meta-analysis, reported in accordance with the Preferred Reporting Items for Systematic Reviews and Meta-Analyses (PRISMA) 2020 guidance, of prospective trials of B-cell maturation antigen (BCMA)- or G protein-coupled receptor class C group 5 member D (GPRC5D)-directed CD3 bispecific antibodies in RRMM that reported the objective response rate (ORR) in patients with baseline EMD. One estimate per trial was included; proportions were pooled using a random-effects model on the logit scale with restricted maximum-likelihood estimation of between-study variance, and heterogeneity was assessed with the Cochran Q test and the I-squared statistic; fixed-effect and leave-one-out sensitivity analyses were performed, and risk of bias was appraised for each EMD subgroup. Four prospective studies comprising 144 patients with baseline EMD were included. Study-level ORRs were 58.3% for teclistamab, 38.5% for elranatamab, 52.6% for linvoseltamab, and 44.6% for talquetamab when recommended phase 2 dose cohorts were combined. The random-effects pooled ORR was 45.2% (95% CI, 37.2-53.4), with no observed between-study heterogeneity (I-squared = 0%); estimates were identical under a fixed-effect model, and leave-one-out pooled ORRs ranged narrowly from 44.0% to 47.6%. BCMA- and GPRC5D-directed bispecific antibodies produce objective responses in approximately half of patients with RRMM and baseline EMD, with broadly similar activity across agents despite high-risk biology, although the small number of trials and their differing, sometimes paramedullary-inclusive, definitions of EMD warrant caution in interpreting this estimate. These pooled estimates provide a benchmark for patient counseling and trial design and support combination strategies to improve outcomes in this population.

## Introduction and background

Multiple myeloma is characterized by the clonal proliferation of malignant plasma cells within the bone marrow. In a subset of patients, however, myeloma cells acquire the capacity to grow independently of the marrow microenvironment and form soft-tissue tumors. True extramedullary disease (EMD) denotes plasmacytomas that develop via hematogenous dissemination and remain noncontiguous with bone; it is documented in roughly 7% to 18% of patients at diagnosis and in as many as 20% at relapse [1,2]. Biologically, EMD is aggressive, is enriched for high-risk cytogenetic abnormalities and genomic instability, and forms a spatially complex, solid-tumor-like microenvironment from which effector T cells are often excluded or in which they become dysfunctional [2-4].

Clinically, patients with EMD consistently experience markedly lower response rates and substantially shorter progression-free and overall survival than those with marrow-confined disease, and this unfavorable prognosis has persisted through successive treatment eras, including the advent of proteasome inhibitors, immunomodulatory drugs, and anti-CD38 monoclonal antibodies [5-8].

In recent years, bispecific antibodies have become highly active treatments for relapsed or refractory multiple myeloma (RRMM). B-cell maturation antigen (BCMA) and G protein-coupled receptor class C group 5 member D (GPRC5D) are cell-surface proteins expressed at high levels and with relative selectivity on malignant plasma cells, which makes them attractive targets for immunotherapy. Bispecific antibodies engage these targets by binding the tumor antigen with one arm and the CD3 receptor on a patient's own T cells with the other, physically bridging T cells to myeloma cells and triggering T-cell-mediated tumor cell killing. In pivotal trials of heavily pretreated, triple-class-exposed patients, the BCMA-directed agents teclistamab, elranatamab, and linvoseltamab and the GPRC5D-directed agent talquetamab produced overall response rates of approximately 52% to 71%, leading to regulatory approval and rapid clinical adoption [9-13].

However, subgroup analyses across these trials have repeatedly suggested attenuated responses among patients with baseline EMD. These subgroups are typically small, are defined inconsistently from one trial to the next, and are often reported only as exploratory, descriptive analyses, which makes the individual estimates difficult to interpret and motivates a quantitative synthesis [9-13]. Prior meta-analyses have quantified the poor outcomes of EMD treated with standard and earlier novel-agent regimens [8], but the expected activity of modern bispecific antibody monotherapy in patients with baseline EMD has not been systematically synthesized.

We therefore conducted a systematic review and meta-analysis to estimate the pooled objective response rate (ORR), defined as the proportion of patients achieving a partial response or better, to BCMA- and GPRC5D-directed bispecific antibodies in patients with RRMM and baseline EMD. ORR was chosen as the primary outcome because it is a standardized, objectively defined efficacy measure that is consistently reported across these trials and is the endpoint most reliably available for the EMD subgroup.

## Review

Methods

Protocol and Reporting

This systematic review and meta-analysis was conducted and reported in accordance with the Preferred Reporting Items for Systematic Reviews and Meta-Analyses (PRISMA) 2020 guidance [14]. The review was not registered, and a protocol was not separately published. A completed PRISMA 2020 checklist accompanies the submission as Table A2 in the Appendices.

Search Strategy and Eligibility

PubMed, the ClinicalTrials.gov trial registry, and Google Scholar were searched from inception to June 2026 to identify prospective clinical trials of BCMA-CD3 or GPRC5D-CD3 bispecific antibodies in RRMM. The full search strategy for each source, including the terms and Boolean operators applied, is provided in Table 1. For Google Scholar, the first 200 records ranked by relevance were screened to capture conference abstracts and other grey literature. Reference lists of eligible trials and pertinent reviews were additionally screened by hand. Studies were assessed against prespecified inclusion and exclusion criteria, which are listed individually in Table 2. In brief, a prospective trial was eligible if it reported an ORR that could be extracted specifically for patients with baseline EMD, whereas retrospective or real-world series, narrative reviews, preclinical studies, combination-regimen studies, and reports without an extractable EMD-specific response rate were not eligible.

**Table 1:** Literature search strategy. Searches were run from database inception to June 2026 with no language or date restrictions and identified 2,858 records (PubMed, n = 1,950; ClinicalTrials.gov, n = 708; Google Scholar, n = 200). Eligibility was restricted to prospective clinical trials reporting an extractable objective response rate in patients with baseline extramedullary disease. BCMA: B-cell maturation antigen; GPRC5D: G protein-coupled receptor class C group 5 member D.

Source	Search terms	Filters	Date last searched
PubMed	"multiple myeloma" AND ("bispecific antibody" OR "BCMA" OR "GPRC5D" OR "teclistamab" OR "elranatamab" OR "linvoseltamab" OR "talquetamab")	None	June 2026
ClinicalTrials.gov	Condition: "multiple myeloma"; other terms: bispecific antibody, BCMA, GPRC5D, teclistamab, elranatamab, linvoseltamab, talquetamab	Interventional studies	June 2026
Google Scholar	Same keyword combination; first 200 records ranked by relevance	None	June 2026
Reference lists	Reference lists of eligible studies and relevant reviews	Not applicable	June 2026

**Table 2:** Inclusion and exclusion criteria. Each row lists a single inclusion or exclusion criterion. BCMA: B-cell maturation antigen; GPRC5D: G protein-coupled receptor class C group 5 member D.

Inclusion or exclusion	Criterion
Inclusion	Prospective clinical trial
Inclusion	Evaluated a BCMA-CD3 or GPRC5D-CD3 bispecific antibody as monotherapy
Inclusion	Enrolled patients with relapsed or refractory multiple myeloma
Inclusion	Reported an extractable objective response rate in patients with baseline extramedullary disease
Exclusion	Retrospective or real-world observational study
Exclusion	Review article, meta-analysis, editorial, or commentary
Exclusion	Preclinical or translational study without a clinical extramedullary-disease response rate
Exclusion	Bispecific antibody evaluated only in combination with another antimyeloma agent
Exclusion	No extractable extramedullary-disease-specific response rate
Exclusion	Duplicate or overlapping report of an already-included cohort

Study Selection

**Figure 1:**
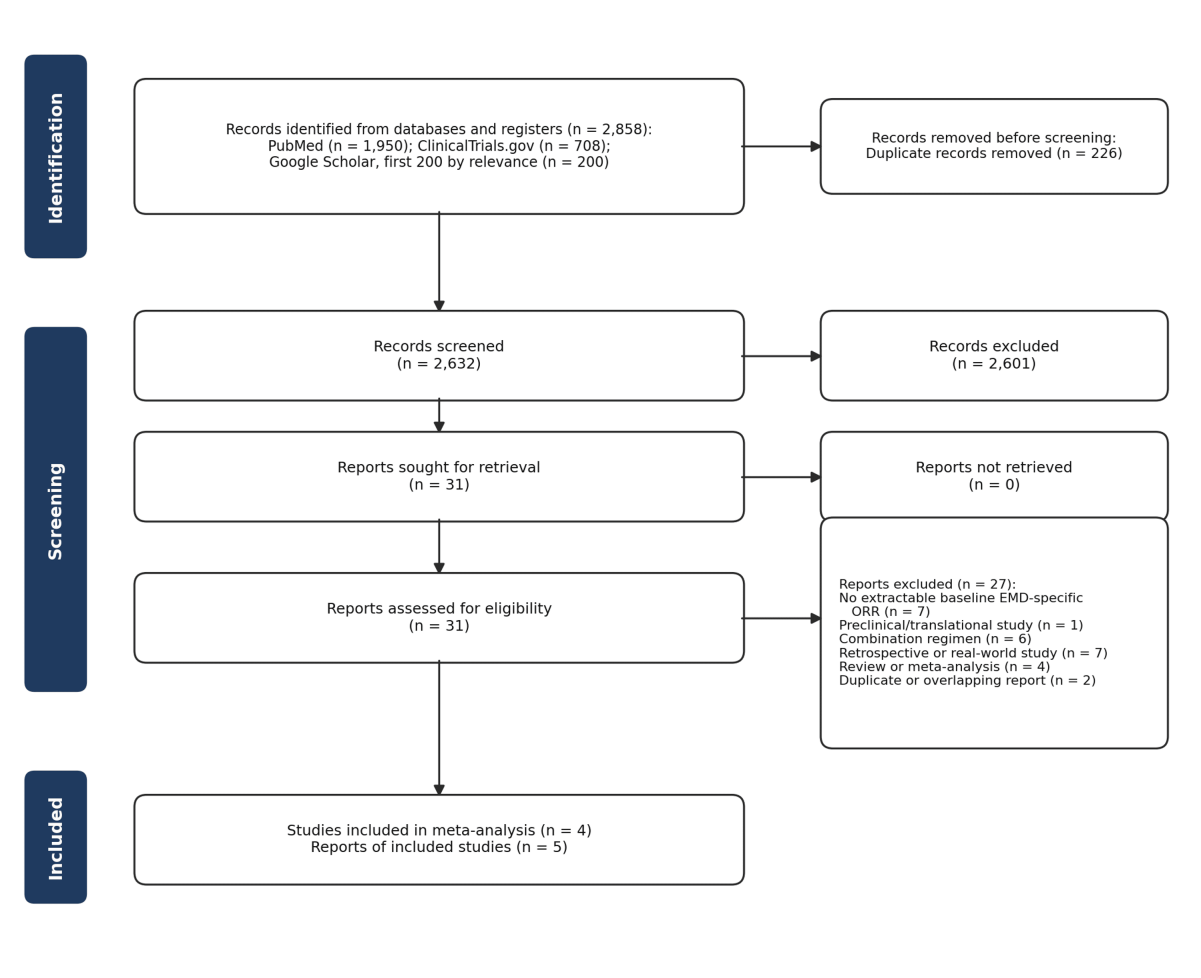
PRISMA 2020 flow diagram of study identification, screening, and inclusion. EMD: extramedullary disease; ORR: objective response rate; PRISMA: Preferred Reporting Items for Systematic Reviews and Meta-Analyses.

After duplicate records were removed, titles and abstracts were screened against the eligibility criteria, and the full texts of potentially eligible records were assessed, each step performed independently by two authors, with disagreements resolved by discussion and, where needed, adjudication by a third author. The study selection process is summarized in Figure 1.

Definitions and Data Extraction

EMD was defined according to individual study criteria. These definitions were not uniform across trials: they ranged from soft-tissue plasmacytomas strictly noncontiguous with bone (true EMD) to broader definitions that also encompassed paramedullary lesions with a soft-tissue component, and one trial additionally required a minimum lesion size of 2 cm. The trial-specific definitions, together with the prior BCMA-directed exposure of each cohort, are summarized in Table A1 in the Appendices. Objective response was defined as partial response or better according to International Myeloma Working Group criteria [15]. For each study, the number of patients with baseline EMD and the number achieving an objective response were extracted. These counts were taken directly from each trial's primary publication and checked against the source reports; the original investigators were not contacted, as the EMD-specific data were available in the published reports. When trials reported multiple dose cohorts, recommended phase 2 dose cohorts were combined to generate a single estimate per trial, preserving statistical independence.

Risk of Bias Assessment

As the included data were single-arm response proportions reported within prospective trials rather than comparative effect estimates, methodological quality and risk of bias were appraised using domains adapted from the National Heart, Lung, and Blood Institute's Quality Assessment Tool for Case Series Studies, applied to the EMD subgroup of each trial [16]. This tool was selected because the included data were single-arm response proportions reported within prospective trials rather than comparative effect estimates, for which randomized-trial risk-of-bias instruments are not designed. The domains assessed were the representativeness of the EMD subgroup within a clearly defined, prospectively followed trial population; the objectivity and reproducibility of the EMD definition; whether the EMD subgroup analysis was prespecified rather than post hoc; the use of standardized International Myeloma Working Group response criteria; and the precision afforded by the subgroup sample size. Each domain was rated as low, moderate, or high concern, and an overall judgment was assigned for each study. Risk of bias was assessed by the same reviewers who performed study selection, with disagreements resolved by discussion.

Statistical Analysis

BCMA- and GPRC5D-directed agents were analyzed together because all are CD3-engaging bispecific antibodies that elicit objective responses through the same mechanism of T-cell redirection, providing an overall benchmark for the class; study-level results for each agent are also reported separately. Each observed response proportion was logit-transformed and combined under a random-effects model, with the between-study variance estimated by restricted maximum likelihood; the pooled result was back-transformed to the proportion scale for reporting. For individual studies, 95% confidence intervals (CIs) were derived using the exact (Clopper-Pearson) binomial method. Heterogeneity was evaluated with the Cochran Q and the I-squared statistic. As sensitivity analyses, random-effects and fixed-effect models were compared, and a leave-one-out procedure was applied that omitted each study in turn. All computations were carried out in R (R Foundation for Statistical Computing, Vienna, Austria) with the metafor package. The data extracted for the meta-analysis are presented in Results, and the analysis code is available from the corresponding author on reasonable request.

Results

Study Selection and Characteristics

The search identified 2,858 records (1,950 from PubMed, 708 from ClinicalTrials.gov, and 200 from Google Scholar). After 226 duplicates were removed, 2,632 records were screened, and 2,601 were excluded; 31 full-text reports were then assessed for eligibility, of which 27 were excluded (cited with reasons in Table A3 in the Appendices), and four prospective studies met the inclusion criteria, comprising 144 patients with baseline EMD (Figure 1). Three studies evaluated BCMA-directed bispecific antibodies, namely, teclistamab (MajesTEC-1 cohort C, post-BCMA), elranatamab (MagnetisMM-3 cohort A), and linvoseltamab (LINKER-MM1), and one study evaluated the GPRC5D-directed agent talquetamab (MonumenTAL-1) [9-13]. For talquetamab, the recommended phase 2 dose cohorts (0.4 mg/kg weekly and 0.8 mg/kg every two weeks) were combined to yield a single estimate. All studies enrolled heavily pretreated populations with a median of five to six prior lines of therapy. Notably, the trials differed both in how EMD was defined and in prior exposure to BCMA-directed therapy: the teclistamab estimate was derived from a post-BCMA cohort, whereas the elranatamab cohort was naive to BCMA-directed therapy (Table A1 in the Appendices). Study-level data are presented in Table 3.

**Table 3:** Study-level objective response rates in patients with baseline extramedullary disease. BCMA: B-cell maturation antigen; CI: confidence interval; GPRC5D: G protein-coupled receptor class C group 5 member D; ORR: objective response rate. Study-level 95% CIs are exact (Clopper-Pearson) binomial intervals. The pooled estimate is from a random-effects model on the logit scale (restricted maximum likelihood), back-transformed to the proportion scale (I-squared = 0%; Cochran Q = 1.96, df = 3, P = 0.58; 95% prediction interval = 28%-63%).

Study (trial)	Target	Responders/total	ORR (%)	95% CI (%)
Teclistamab (MajesTEC-1 cohort C, post-BCMA) [9,10]	BCMA	7/12	58.3	27.7-84.8
Elranatamab (MagnetisMM-3 cohort A) [11]	BCMA	15/39	38.5	23.4-55.4
Linvoseltamab (LINKER-MM1) [12]	BCMA	10/19	52.6	28.9-75.6
Talquetamab (MonumenTAL-1; weekly + every 2 weeks) [13]	GPRC5D	33/74	44.6	33.0-56.6
Random-effects pooled estimate	BCMA and GPRC5D	65/144	45.2	37.2-53.4

Risk of Bias

Overall risk of bias was low for the elranatamab subgroup and moderate for the teclistamab, linvoseltamab, and talquetamab subgroups (Table 4). All four trials enrolled clearly defined, prospectively followed populations and assessed response using standardized International Myeloma Working Group criteria [15]. The principal concerns were the small size of the EMD subgroups, which limited precision, most notably for teclistamab (n = 12) and linvoseltamab (n = 19); the heterogeneous EMD definitions, including the broader, paramedullary-inclusive definition applied for elranatamab; and the post hoc, exploratory nature of the talquetamab EMD analysis. No study was judged to be at high overall risk of bias, although the consistently small subgroup sizes temper confidence in the individual estimates.

**Table 4:** Risk of bias assessment of the extramedullary disease subgroup in each included trial. Risk of bias was appraised using domains adapted from the National Heart, Lung, and Blood Institute's Quality Assessment Tool for Case Series Studies [16], applied to each trial's EMD subgroup; each domain was rated as low, moderate, or high concern. EMD subgroup sizes were 12 (teclistamab), 39 (elranatamab), 19 (linvoseltamab), and 74 (talquetamab). EMD: extramedullary disease; IMWG: International Myeloma Working Group.

Study (trial; cohort)	Prospective cohort	Objective EMD definition	Prespecified subgroup	IMWG criteria	Precision (size)	Overall risk of bias
Teclistamab (MajesTEC-1 cohort C) [9,10]	Low	Low	Moderate	Low	High	Moderate
Elranatamab (MagnetisMM-3 cohort A) [11]	Low	Moderate	Low	Low	Moderate	Low
Linvoseltamab (LINKER-MM1) [12]	Low	Low	Low	Low	High	Moderate
Talquetamab (MonumenTAL-1) [13]	Low	Moderate	High	Low	Low	Moderate

Pooled Objective Response Rate

**Figure 2:**
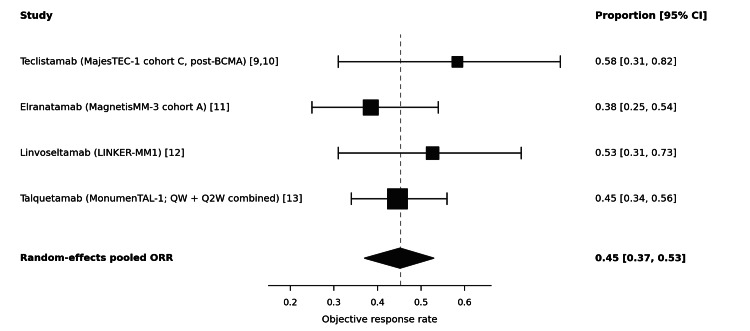
Forest plot of objective response rate in patients with baseline extramedullary disease by bispecific antibody (one estimate per study), with the random-effects pooled estimate. Squares represent study-level proportions, and the diamond represents the pooled estimate; the dashed vertical line denotes the pooled benchmark (0.453). CI: confidence interval; ORR: objective response rate.

Across the four studies, the random-effects pooled ORR in patients with baseline EMD was 45.2% (95% CI, 37.2-53.4), with no observed between-study heterogeneity (I-squared = 0%; Cochran Q = 1.96, df = 3, P = 0.58) (Figure 2). The 95% prediction interval was 28% to 63%. With only four contributing studies, however, statistical power to detect heterogeneity was limited, and the absence of observed heterogeneity should be interpreted with caution rather than as firm evidence of consistency across agents.

Individual Study Estimates

Study-level ORRs in patients with baseline EMD were 58.3% (7/12) for teclistamab, 38.5% (15/39) for elranatamab, 52.6% (10/19) for linvoseltamab, and 44.6% (33/74) for talquetamab when recommended phase 2 dose cohorts were combined. Among the BCMA-directed agents, ORRs ranged from 38.5% to 58.3%, compared with 44.6% for the single GPRC5D-directed agent; with only one GPRC5D-directed study, a formal comparison between antigen targets was not possible.

Sensitivity Analyses

Fixed-effect and random-effects models produced identical pooled estimates. Leave-one-out analysis produced pooled ORRs ranging narrowly from 44.0% to 47.6%, with overlapping confidence intervals, indicating that the overall result was not driven by any single study.

Discussion

Principal Findings

In this meta-analysis of four prospective trials and 144 patients, BCMA- and GPRC5D-directed bispecific antibodies achieved objective responses in approximately 45% of patients with RRMM and baseline EMD. Activity appeared broadly similar across agents and antigen targets; although no between-study heterogeneity was detected, the small number of trials limits the certainty of this finding, and the pooled estimate was robust to modeling assumptions and to the sequential exclusion of individual studies.

These response rates are clinically meaningful in a population with historically poor outcomes, yet they are appreciably lower than the overall response rates of approximately 52% to 71% reported for the same agents in their broader trial populations [9-13]. The convergence of estimates across BCMA- and GPRC5D-directed agents suggests that the choice of tumor antigen alone is insufficient to overcome the adverse biology of EMD.

Biological Explanations

Several features of EMD may explain this attenuated response to T-cell-redirecting therapy. EMD lesions display a spatially organized, solid-tumor-like architecture in which cytotoxic T cells are often confined to niches segregated from malignant plasma cells or adopt an exhausted phenotype, limiting effective engagement at the tumor site [3,4]. EMD is further marked by pronounced subclonal genomic heterogeneity and spatially variable expression of the target antigens BCMA and GPRC5D, which may permit antigen-low immune escape [3,4].

Clinical Implications and Future Directions

These data provide a quantitative benchmark to counsel patients with EMD regarding the expected likelihood of response to bispecific monotherapy and to inform the design and interpretation of future trials. They also support the rationale for intensified strategies in this population. Dual-antigen targeting with combined BCMA- and GPRC5D-directed bispecific antibodies has shown markedly higher activity in a dedicated EMD cohort: the phase 2 RedirecTT-1 study reported an ORR of approximately 79% with talquetamab plus teclistamab in patients with true EMD [17], substantially exceeding the monotherapy benchmark established here. Together with the spatial antigen heterogeneity and immune exclusion that characterize EMD lesions, these results provide a strong rationale for prospectively evaluating dual-targeting regimens, as well as cytoreductive (debulking) and other combination approaches, specifically in patients with EMD. More broadly, because patients with EMD are consistently underrepresented in registrational trials and are usually captured only in post hoc subgroup analyses, future studies should prospectively stratify randomization by EMD status or enroll dedicated EMD-enriched cohorts. Adoption of a uniform, imaging-based definition of EMD, supported by high-sensitivity functional imaging such as fluorodeoxyglucose positron emission tomography and by harmonized assessment of soft-tissue responses, would further enable reliable comparison across trials and strengthen the evidence base for this high-risk population.

Comparison With Previous Work

Our findings extend prior meta-analytic work, which established the adverse prognosis of EMD treated with standard and earlier novel-agent regimens [8], into the contemporary era of T-cell-redirecting bispecific antibodies, confirming that EMD remains a high-risk feature even with these highly active agents.

Interpretation of the pooled estimate is further complicated by differences in prior BCMA-directed exposure across the included cohorts. The teclistamab estimate was derived from MajesTEC-1 cohort C, which exclusively enrolled patients previously treated with BCMA-directed therapy (an antibody-drug conjugate and/or chimeric antigen receptor T cells), whereas the elranatamab estimate came from a cohort naive to BCMA-directed therapy. Prior exposure to BCMA-directed agents can attenuate the activity of subsequent BCMA-directed bispecific antibodies through several mechanisms, including downregulation or biallelic loss of the target antigen (TNFRSF17), acquired extracellular-domain mutations that impair antibody binding despite preserved surface antigen expression, and diminished T-cell fitness after repeated immune engagement [18]. Although teclistamab yielded the highest observed response rate in this analysis despite enrolling a post-BCMA population, this finding likely reflects its small sample size and the strict true-EMD definition applied in that cohort rather than genuinely superior activity, highlighting the need for caution when making cross-trial comparisons in this setting.

Limitations

This analysis has several limitations. First, the operational definition of EMD differed across the included trials, ranging from plasmacytomas strictly noncontiguous with bone to broader definitions that also captured paramedullary lesions, with one trial imposing a minimum 2 cm size threshold (Table A1 in the Appendices); this definitional heterogeneity limits the comparability of EMD subgroups across studies and the interpretation of the pooled estimate. Second, the included cohorts differed in prior BCMA-directed exposure, which may independently influence response and confound direct comparison between agents, particularly given the inclusion of the post-BCMA teclistamab cohort. Third, only study-level rather than patient-level data were available, precluding adjustment for confounders such as cytogenetic risk and tumor burden. Fourth, the small number of eligible studies and the limited size of the EMD subgroups reduced precision, precluded formal statistical comparison between antigen targets, and substantially limited the power to detect between-study heterogeneity, such that the observed I-squared of 0% does not exclude clinically meaningful variation across trials. Fifth, our search was restricted to PubMed, the ClinicalTrials.gov registry, and Google Scholar; Embase, Scopus, Web of Science, and the Cochrane Library were not accessible to the authors, and studies indexed only in those databases may have been missed. Finally, with only four included studies, meta-regression to explore sources of heterogeneity and formal assessment of publication bias were not feasible. In addition, the ORR was the only outcome consistently reported for the baseline-EMD subgroups; progression-free survival, overall survival, duration of response, and complete or very good partial response rates were not available specifically for these subgroups in most trials and could not be synthesized.

## Conclusions

Bispecific antibodies directed against BCMA or GPRC5D produce objective responses in approximately half of patients with RRMM and baseline EMD, with broadly similar activity across the agents studied. Because these estimates derive from a small number of trials that defined EMD differently, they should be regarded as an approximate benchmark for patient counseling and trial design rather than a definitive measure of efficacy. They underscore the need for combination and other intensified strategies, and for prospective studies that use uniform EMD definitions, to improve outcomes in this difficult-to-treat population.

## Appendices

Table A1

**Table 5:** Definitions of extramedullary disease and prior BCMA-directed exposure across the included trials. BCMA: B-cell maturation antigen; CAR: chimeric antigen receptor; EMD: extramedullary disease; GPRC5D: G protein-coupled receptor class C group 5 member D; ORR: objective response rate; Q2W: every two weeks; QW: once weekly; RP2D: recommended phase 2 dose. Definitions are reproduced as reported in each trial's primary publication; bracketed numbers refer to references in the main manuscript. The teclistamab and linvoseltamab cohorts used true-EMD definitions (lesions not contiguous with bone), whereas the elranatamab cohort used a broader definition that also included paramedullary lesions, and the talquetamab report did not fully specify bone-independence or a size threshold. These differences, together with differing prior BCMA-directed exposure, limit direct comparison of EMD subgroups across trials.

Bispecific antibody (trial; cohort)	Antigen target	EMD definition as reported	Paramedullary lesions included	Minimum lesion size	Prior BCMA-directed therapy in the cohort	EMD subgroup (responders/total; ORR)
Teclistamab (MajesTEC-1, cohort C) [9,10]	BCMA	≥1 extramedullary soft-tissue plasmacytoma not associated with bone (true EMD)	No	Not specified	Yes (all; prior antibody-drug conjugate and/or CAR T-cell therapy)	7/12; 58.3%
Elranatamab (MagnetisMM-3, cohort A) [11]	BCMA	Presence of any plasmacytoma, extramedullary and/or paramedullary with a soft-tissue component (assessed by blinded independent central review)	Yes	Not specified	No (BCMA-naive cohort)	15/39; 38.5%
Linvoseltamab (LINKER-MM1, 200 mg) [12]	BCMA	Non-bone-associated plasmacytoma ≥2 cm (paramedullary lesions excluded)	No	≥2 cm	Not defined by prior BCMA exposure	10/19; 52.6%
Talquetamab (MonumenTAL-1, RP2D; QW + Q2W combined) [13]	GPRC5D	≥1 extramedullary (soft-tissue) plasmacytoma at baseline (further specification of bone-independence and size not detailed in the primary report)	Not specified	Not specified	RP2D cohorts naive to prior T-cell-redirection therapy	33/74; 44.6%

Table A2

**Table 6:** PRISMA 2020 checklist. Reporting follows the PRISMA 2020 statement [14]. Locations refer to sections of the accompanying manuscript.

Section and topic	Item #	Checklist item	Location where the item is reported
TITLE			
Title	1	Identify the report as a systematic review.	Title
ABSTRACT			
Abstract	2	See the PRISMA 2020 for Abstracts checklist.	Abstract
INTRODUCTION			
Rationale	3	Describe the rationale for the review in the context of existing knowledge.	Introduction (paragraphs 1-4)
Objectives	4	Provide an explicit statement of the objective(s) or question(s) the review addresses.	Introduction (final paragraph)
METHODS			
Eligibility criteria	5	Specify the inclusion and exclusion criteria for the review and how studies were grouped for the syntheses.	Methods, Search strategy and eligibility; Table 2
Information sources	6	Specify all databases, registers, websites, organizations, reference lists, and other sources searched or consulted to identify studies. Specify the date when each source was last searched or consulted.	Methods, Search strategy and eligibility; Table 1 (PubMed, ClinicalTrials.gov, and Google Scholar, searched through June 2026)
Search strategy	7	Present the full search strategies for all databases, registers and websites, including any filters and limits used.	Methods, Search strategy and eligibility; Table 1
Selection process	8	Specify the methods used to decide whether a study met the inclusion criteria of the review, including how many reviewers screened each record and each report retrieved, whether they worked independently, and, if applicable, details of automation tools used in the process.	Methods, Study selection; Figure 1
Data collection process	9	Specify the methods used to collect data from reports, including how many reviewers collected data from each report, whether they worked independently, any processes for obtaining or confirming data from study investigators, and, if applicable, details of automation tools used in the process.	Methods, Definitions and data extraction
Data items	10a	List and define all outcomes for which data were sought. Specify whether all results that were compatible with each outcome domain in each study were sought (e.g., for all measures, time points, analyses), and if not, the methods used to decide which results to collect.	Methods, Definitions and data extraction (objective response rate, partial response or better per IMWG criteria)
Data items	10b	List and define all other variables for which data were sought (e.g., participant and intervention characteristics, funding sources). Describe any assumptions made about any missing or unclear information.	Methods, Definitions and data extraction; Results, Table 3 (agent, antigen target, prior lines of therapy)
Study risk of bias assessment	11	Specify the methods used to assess risk of bias in the included studies, including details of the tool(s) used, how many reviewers assessed each study and whether they worked independently, and, if applicable, details of automation tools used in the process.	Methods, Risk of bias assessment
Effect measures	12	Specify for each outcome the effect measure(s) (e.g., risk ratio, mean difference) used in the synthesis or presentation of results.	Methods, Statistical analysis (objective response rate expressed as a proportion)
Synthesis methods	13a	Describe the processes used to decide which studies were eligible for each synthesis (e.g., tabulating the study intervention characteristics and comparing against the planned groups for each synthesis).	Methods, Search strategy and eligibility; Definitions and data extraction
Synthesis methods	13b	Describe any methods required to prepare the data for presentation or synthesis, such as handling of missing summary statistics or data conversions.	Methods, Statistical analysis (logit transformation; one estimate per trial)
Synthesis methods	13c	Describe any methods used to tabulate or visually display results of individual studies and syntheses.	Methods, Statistical analysis; Results, Table 3 and Figure 2
Synthesis methods	13d	Describe any methods used to synthesize results and provide a rationale for the choice(s). If meta-analysis was performed, describe the model(s), method(s) to identify the presence and extent of statistical heterogeneity, and software package(s) used.	Methods, Statistical analysis (random-effects model on the logit scale, REML; I-squared and Cochran Q; R metafor package)
Synthesis methods	13e	Describe any methods used to explore possible causes of heterogeneity among study results (e.g., subgroup analysis, meta-regression).	Not performed (only four studies; noted in Discussion, Limitations)
Synthesis methods	13f	Describe any sensitivity analyses conducted to assess the robustness of the synthesized results.	Methods, Statistical analysis; Results, Sensitivity analyses (fixed-effect and leave-one-out analyses)
Reporting bias assessment	14	Describe any methods used to assess risk of bias due to missing results in a synthesis (arising from reporting biases).	Not performed (only four studies; acknowledged in Discussion, Limitations)
Certainty assessment	15	Describe any methods used to assess certainty (or confidence) in the body of evidence for an outcome.	Not performed (formal GRADE assessment not conducted)
RESULTS			
Study selection	16a	Describe the results of the search and selection process, from the number of records identified in the search to the number of studies included in the review, ideally using a flow diagram.	Results, Study selection and characteristics; Figure 1
Study selection	16b	Cite studies that might appear to meet the inclusion criteria but were excluded, and explain why they were excluded.	Results, Table 7 (excluded full-text records cited with reasons)
Study characteristics	17	Cite each included study and present its characteristics.	Results, Study selection and characteristics; Table 3; references [9-13]
Risk of bias in studies	18	Present assessments of risk of bias for each included study.	Results, Risk of bias; Table 4
Results of individual studies	19	For all outcomes, present, for each study: (a) summary statistics for each group (where appropriate) and (b) an effect estimate and its precision (e.g., confidence/credible interval), ideally using structured tables or plots.	Results, Individual study estimates; Table 3; Figure 2
Results of syntheses	20a	For each synthesis, briefly summarize the characteristics and risk of bias among contributing studies.	Results, Study selection and characteristics; Table 3
Results of syntheses	20b	Present results of all statistical syntheses conducted. If meta-analysis was done, present for each the summary estimate and its precision (e.g., confidence/credible interval) and measures of statistical heterogeneity.	Results, Pooled objective response rate; Figure 2 (pooled ORR 45.2%, 95% CI 37.2-53.4; I-squared = 0%)
Results of syntheses	20c	Present results of all investigations of possible causes of heterogeneity among study results.	Not applicable (no heterogeneity investigation performed; I-squared = 0%)
Results of syntheses	20d	Present results of all sensitivity analyses conducted to assess the robustness of the synthesized results.	Results, Sensitivity analyses (leave-one-out 44.0-47.6%; identical fixed-effect estimate)
Reporting biases	21	Present assessments of risk of bias due to missing results (arising from reporting biases) for each synthesis assessed.	Not assessed (acknowledged in Discussion, Limitations)
Certainty of evidence	22	Present assessments of certainty (or confidence) in the body of evidence for each outcome assessed.	Not assessed (formal GRADE assessment not conducted)
DISCUSSION			
Discussion	23a	Provide a general interpretation of the results in the context of other evidence.	Discussion (Principal Findings; Biological Explanations; Comparison With Previous Work)
Discussion	23b	Discuss any limitations of the evidence included in the review.	Discussion, Limitations
Discussion	23c	Discuss any limitations of the review processes used.	Discussion, Limitations
Discussion	23d	Discuss implications of the results for practice, policy, and future research.	Discussion (Clinical Implications and Future Directions); Conclusions
OTHER INFORMATION			
Registration and protocol	24a	Provide registration information for the review, including the register name and registration number, or state that the review was not registered.	Methods, protocol, and reporting: the review was not registered
Registration and protocol	24b	Indicate where the review protocol can be accessed, or state that a protocol was not prepared.	Methods, protocol, and reporting: a protocol was not separately published
Registration and protocol	24c	Describe and explain any amendments to information provided at registration or in the protocol.	Not applicable
Support	25	Describe sources of financial or non-financial support for the review, and the role of the funders or sponsors in the review.	Provided through the journal submission and disclosure process
Competing interests	26	Declare any competing interests of review authors.	Provided through the journal submission and disclosure process
Availability of data, code, and other materials	27	Report which of the following are publicly available and where they can be found: template data collection forms; data extracted from included studies; data used for all analyses; analytic code; any other materials used in the review.	Extracted data presented in Table 3; analysis code available from the corresponding author on request (Methods, Statistical analysis; R, metafor)

Table A3

**Table 7:** Full-text records excluded after eligibility assessment, with reasons. The 27 full-text records excluded after eligibility assessment are cited with the reason for exclusion, corresponding to the categories shown in Figure 1. Conference abstracts are indicated. EMD: extramedullary disease; ORR: objective response rate.

No.	Excluded study	Reason for exclusion
1	Bahlis NJ, Costello CL, Raje NS, et al. Nat Med. 2023;29:2570-2576.	No extractable baseline EMD-specific ORR
2	Iida S, Ito S, Yokoyama H, et al. Jpn J Clin Oncol. 2024;54:991-1000.	No extractable baseline EMD-specific ORR
3	Ishida T, Kuroda Y, Matsue K, et al. Int J Hematol. 2025;121:222-231.	No extractable baseline EMD-specific ORR
4	Iida S, Sunami K, Ito S, et al. Int J Hematol. 2025. doi:10.1007/s12185-025-03991-5.	No extractable baseline EMD-specific ORR
5	Miao X, Wu LS, Lin SXW, et al. Target Oncol. 2023;18:667-684.	No extractable baseline EMD-specific ORR
6	Cortes-Selva D, Perova T, Skerget S, et al. Blood. 2024;144:615-628.	No extractable baseline EMD-specific ORR
7	Baines AC, Kanapuru B, Zhao J, et al. Clin Cancer Res. 2024;30:5515-5520.	No extractable baseline EMD-specific ORR
8	Eckmann J, Fauti T, Biehl M, et al. Blood. 2025;145:202-219.	Preclinical or translational study
9	Costa LJ, Bahlis NJ, Perrot A, et al. N Engl J Med. 2026;394:739-752.	Combination regimen
10	Cohen YC, Magen H, Gatt M, et al. N Engl J Med. 2025;392:138-149.	Combination regimen
11	Kumar S, Mateos MV, Ye JC, et al. N Engl J Med. 2026;394:51-61.	Combination regimen
12	Chari A, van de Donk NWCJ, Dholaria B, et al. Blood. 2025. doi:10.1182/blood.2025029360.	Combination regimen
13	Nooka AK, Cochrane T, D'Souza A, et al. Blood. 2024;144(Suppl 1) [abstract].	Combination regimen
14	Quach H, Perrot A, Matous J, et al. Blood. 2025;146(Suppl 1):2282 [abstract].	Combination regimen
15	Steinhardt MJ, Schaefers C, Leypoldt LB, et al. Blood Cancer J. 2025;15:126.	Retrospective or real-world study
16	Afrough A, Dima D, Razzo B, et al. Blood Cancer J. 2026;16:12.	Retrospective or real-world study
17	Merz M, Dima D, Hashmi H, et al. Blood Cancer J. 2024;14:214.	Retrospective or real-world study
18	Mohan M, Monge J, Shah N, et al. Blood Cancer J. 2024;14:35.	Retrospective or real-world study
19	Dima D, Davis JA, Ahmed N, et al. Transplant Cell Ther. 2024;30:308.e1-308.e13.	Retrospective or real-world study
20	Riedhammer C, Bassermann F, Besemer B, et al. Leukemia. 2024;38:365-371.	Retrospective or real-world study
21	Al Hadidi S, Szabo A, Mohan Lal B, et al. Blood Cancer J. 2025;15:196.	Retrospective or real-world study
22	Voorhees PM, Kumar S, Usmani SZ, et al. Ann Hematol. 2025.	Review or meta-analysis
23	Liang X, Wang Y, Luo B, et al. J Immunother Cancer. 2024;12:e010064.	Review or meta-analysis
24	Devasia AJ, Chari A, Lancman G. Blood Cancer J. 2024;14:158.	Review or meta-analysis
25	Liu L, Krishnan A. Haematologica. 2024;109:718-724.	Review or meta-analysis
26	Chari A, Touzeau C, Schinke C, et al. Lancet Haematol. 2025.	Duplicate or overlapping report of an included cohort
27	Lee HC, Zonder JA, Dhodapkar MV, et al. Clin Lymphoma Myeloma Leuk. 2026;26:e201-e212.e8.	Duplicate or overlapping report of an included cohort
